# Techniques for extraction and isolation of natural products: a comprehensive review

**DOI:** 10.1186/s13020-018-0177-x

**Published:** 2018-04-17

**Authors:** Qing-Wen Zhang, Li-Gen Lin, Wen-Cai Ye

**Affiliations:** 1 State Key Laboratory of Quality Research in Chinese Medicine, Institute of Chinese Medical Sciences, University of Macau, Macao, People’s Republic of China; 2 Institute of Traditional Chinese Medicine & Natural Products, and Guangdong Provincial Engineering Research Center for Modernization of TCM, College of Pharmacy, Jinan University, Guangzhou, 510632 People’s Republic of China

**Keywords:** Natural products, Extraction, Isolation, Natural medicine, Chromatography, Phytochemical investigation

## Abstract

Natural medicines were the only option for the prevention and treatment of human diseases for thousands of years. Natural products are important sources for drug development. The amounts of bioactive natural products in natural medicines are always fairly low. Today, it is very crucial to develop effective and selective methods for the extraction and isolation of those bioactive natural products. This paper intends to provide a comprehensive view of a variety of methods used in the extraction and isolation of natural products. This paper also presents the advantage, disadvantage and practical examples of conventional and modern techniques involved in natural products research.

## Background

Natural medicines, such as traditional Chinese medicine (TCM) and Ayurveda, were formed and developed in the daily life of ancient people and in the process of their fight against diseases over thousands of years, and they have produced a positive impact on the progress of human civilization. Today, natural medicines not only provide the primary health-care needs for the majority of the population in developing countries but have attracted more and more attention in developed countries due to soaring health-care costs and universal financial austerity. In the USA, approximately 49% of the population has tried natural medicines for the prevention and treatment of diseases [1]. Chemicals known to have medicinal benefits are considered to be “active ingredients” or “active principles” of natural medicines. Natural products have provided the primary sources for new drug development. From the 1940s to the end of 2014, nearly half of the FDA approved chemical drugs for the treatment of human diseases were derived from or inspired by natural products [2, 3]. Natural products offer more drug-like features to molecules from combinatorial chemistry in terms of functional groups, chirality, and structural complexity [4, 5].

The amounts of active ingredients in natural medicines are always fairly low. The lab-intensive and time-consuming extraction and isolation process has been the bottle neck of the application of natural products in drug development. There is an urgent need to develop effective and selective methods for the extraction and isolation of bioactive natural products. This review intends to provide a comprehensive view of a variety of methods used in the extraction and isolation of natural products.

## Extraction

Extraction is the first step to separate the desired natural products from the raw materials. Extraction methods include solvent extraction, distillation method, pressing and sublimation according to the extraction principle. Solvent extraction is the most widely used method. The extraction of natural products progresses through the following stages: (1) the solvent penetrates into the solid matrix; (2) the solute dissolves in the solvents; (3) the solute is diffused out of the solid matrix; (4) the extracted solutes are collected. Any factor enhancing the diffusivity and solubility in the above steps will facilitate the extraction. The properties of the extraction solvent, the particle size of the raw materials, the solvent-to-solid ration, the extraction temperature and the extraction duration will affect the extraction efficiency [6–10].

The selection of the solvent is crucial for solvent extraction. Selectivity, solubility, cost and safety should be considered in selection of solvents. Based on the law of similarity and intermiscibility (like dissolves like), solvents with a polarity value near to the polarity of the solute are likely to perform better and vice versa. Alcohols (EtOH and MeOH) are universal solvents in solvent extraction for phytochemical investigation.

Generally, the finer the particle size is, the better result the extraction achieves. The extraction efficiency will be enhanced by the small particle size due to the enhanced penetration of solvents and diffusion of solutes. Too fine particle size, however, will cost the excessive absorption of solute in solid and difficulty in subsequent filtration.

High temperatures increase the solubility and diffusion. Temperatures that too high, however, may cause solvents to be lost, leading to extracts of undesirable impurities and the decomposition of thermolabile components.

The extraction efficiency increases with the increase in extraction duration in a certain time range. Increasing time will not affect the extraction after the equilibrium of the solute is reached inside and outside the solid material.

The greater the solvent-to-solid ratio is, the higher the extraction yield is; however, a solvent-to-solid ratio that is too high will cause excessive extraction solvent and requires a long time for concentration.

The conventional extraction methods, including maceration, percolation and reflux extraction, usually use organic solvents and require a large volume of solvents and long extraction time. Some modern or greener extraction methods such as super critical fluid extraction (SFC), pressurized liquid extraction (PLE) and microwave assisted extraction (MAE), have also been applied in natural products extraction, and they offer some advantages such as lower organic solvent consumption, shorter extraction time and higher selectivity. Some extraction methods, however, such as sublimation, expeller pressing and enfleurage are rarely used in current phytochemical investigation and will not discussed in this review. A brief summary of the various extraction methods used for natural products is shown in Table 1.

**Table 1:** A brief summary of various extraction methods for natural products

Method	Solvent	Temperature	Pressure	Time	Volume of organic solvent consumed	Polarity of natural products extracted
Maceration	Water, aqueous and non-aqueous solvents	Room temperature	Atmospheric	Long	Large	Dependent on extracting solvent
Percolation	Water, aqueous and non-aqueous solvents	Room temperature, occasionally under heat	Atmospheric	Long	Large	Dependent on extracting solvent
Decoction	Water	Under heat	Atmospheric	Moderate	None	Polar compounds
Reflux extraction	Aqueous and non-aqueous solvents	Under heat	Atmospheric	Moderate	Moderate	Dependent on extracting solvent
Soxhlet extraction	Organic solvents	Under heat	Atmospheric	Long	Moderate	Dependent on extracting solvent
Pressurized liquid extraction	Water, aqueous and non-aqueous solvents	Under heat	High	Short	Small	Dependent on extracting solvent
Supercritical fluid extraction	Supercritical fluid (usually S-CO_2_), sometimes with modifier	Near room temperature	High	Short	None or small	Nonpolar to moderate polar compounds
Ultrasound assisted extraction	Water, aqueous and non-aqueous solvents	Room temperature, or under heat	Atmospheric	Short	Moderate	Dependent on extracting solvent
Microwave assisted extraction	Water, aqueous and non-aqueous solvents	Room temperature	Atmospheric	Short	None or moderate	Dependent on extracting solvent
Pulsed electric field extraction	Water, aqueous and non-aqueous solvents	Room temperature, or under heat	Atmospheric	Short	Moderate	Dependent on extracting solvent
Enzyme assisted extraction	Water, aqueous and non-aqueous solvents	Room temperature, or heated after enzyme treatment	Atmospheric	Moderate	Moderate	Dependent on extracting solvent
Hydro distillation and steam distillation	Water	Under heat	Atmospheric	Long	None	Essential oil (usually non-polar)

### Maceration

This is a very simple extraction method with the disadvantage of long extraction time and low extraction efficiency. It could be used for the extraction of thermolabile components.

**Fig. 1:**
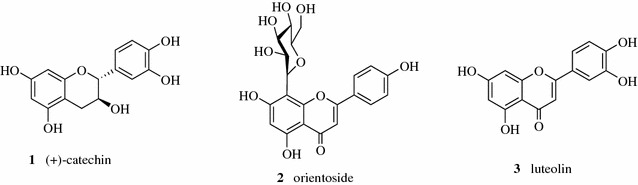
Structures of compounds **1**–**3**

Ćujić et al. achieved high yields of total phenols and total anthocyanins from chokeberry fruit at an optimized condition with 50% ethanol, a solid–solvent ratio of 1:20 and particle size of 0.75 mm, which suggested that maceration was a simple and effective method for the extraction of phenolic compounds from chokeberry fruit [11]. A study on the extraction of catechin (**1**, Fig. 1) from *Arbutus unedo* L. fruits using maceration, microwave-assisted and ultrasound extraction techniques showed that microwave-assisted extraction (MAE) was the most effective, but a lower temperature was applied in maceration with nearly identical extraction yields, which can be translated into economic benefits [12]. Jovanović et al. evaluated the extraction efficiency of polyphenols from *Serpylli herba* using various extraction techniques (maceration, heat assisted extraction and ultrasonic-assisted extraction). Based on the content of total polyphenols, ultrasonic-assisted extraction produced the highest total flavonoids yield and no statistically significant difference were found between maceration and heat assisted extraction [13]. *Cajanus cajan* leaves are used in Chinese folk medicine for the treatment of hepatitis, chickenpox and diabetes. Flavonoids are the bioactive compounds. Jin et al. compared extraction rates of orientoside (**2**), luteolin (**3**), and total flavonoids from *C. cajan* leaves by microwave-assisted method, reflux extraction, ultrasound-assisted extraction, and maceration extraction. The extraction efficiency of orientoside, luteolin, and total flavonoids was found to be the lowest in the extract from maceration method [14].

### Percolation

Percolation is more efficient than maceration because it is a continuous process in which the saturated solvent is constantly being replaced by fresh solvent.

**Fig. 2:**
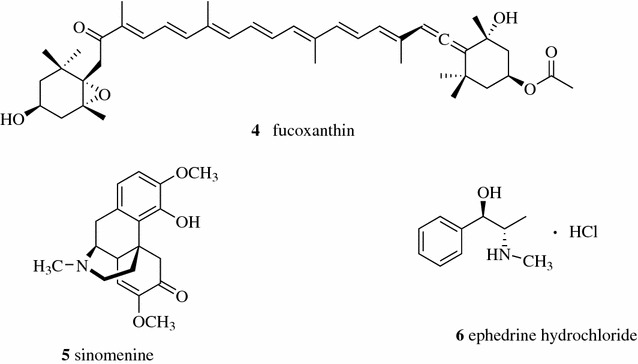
Structures of compounds **4**–**6**

Zhang et al. compared the percolation and refluxing extraction methods to extract *Undaria pinnatifida*. They found that the contents of the major component, fucoxanthin (**4**, Fig. 2), from the percolation extraction method was higher than that from the refluxing method while there was no significant difference in extract yield between the two methods [15]. Goupi patch is a compound Chinese medicine preparation consisting of 29 Chinese medicines. Fu et al. used the whole alkaloids content determined by acid–base titration as the index and optimized the ethanol percolation method as soaking the medicine with 55% alcohol for 24 h and then percolating with 12 times the amount of 55% alcohol [16]. When using the extracting rate of sinomenine (**5**) and ephedrine hydrochloride (**6**) as the index, Gao developed another optimized percolation method: soaking the medicine with 70% ethanol for 24 h and then percolating with 20 times the amount of 70% ethanol. The transfer rates of sinomenine and ephedrine hydrochloride were 78.23 and 76.92%, respectively [17].

### Decoction

The extract from decoction contains a large amount of water-soluble impurities. Decoction cannot be used for the extraction of thermolabile or volatile components.

**Fig. 3:**
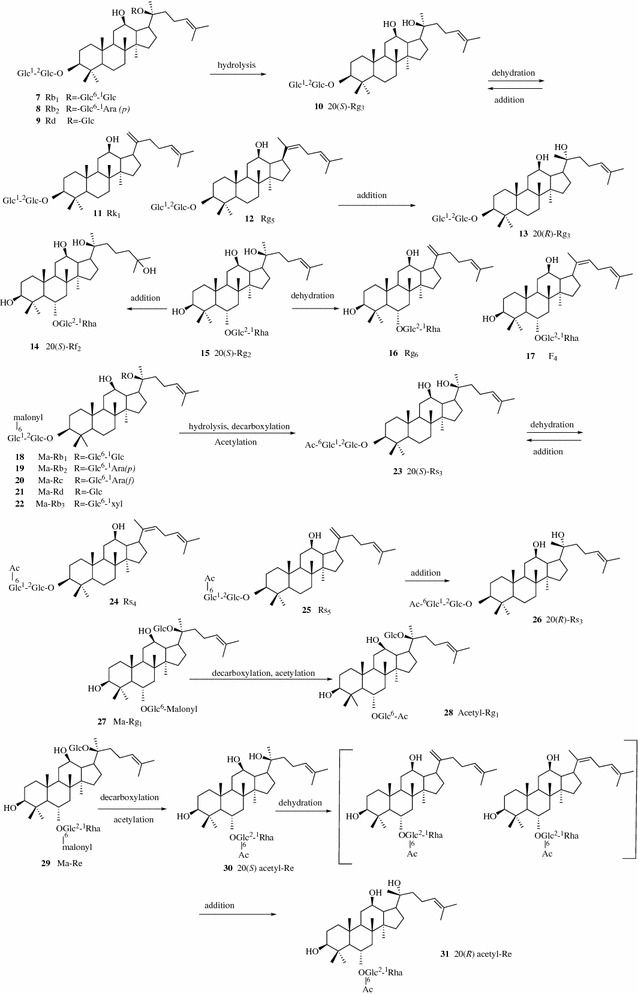
Possible mechanisms of the chemical conversion of ginsenosides (**7**–**31**) in decoction

**Fig. 4:**
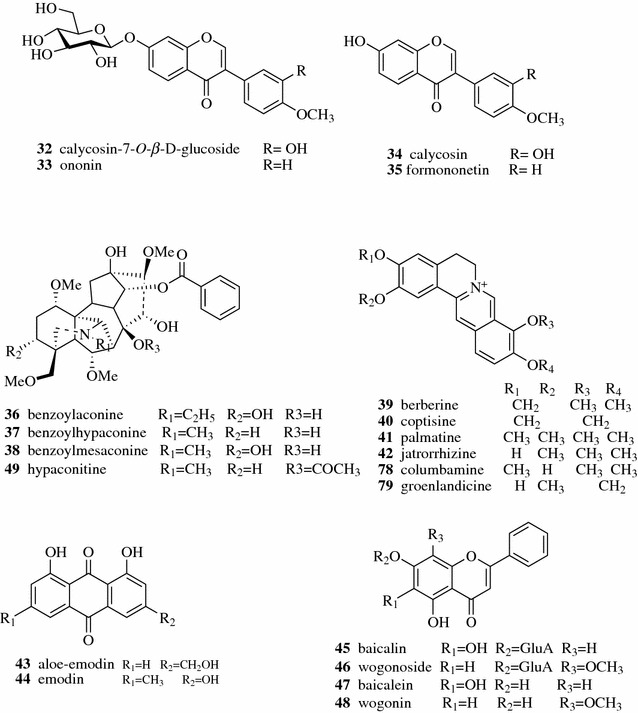
Structures of compounds **32**–**48** and **78**–**79**

The ginsenosides (**7**–**31**) in ginseng encounter hydrolysis, dehydration, decarboxylation and addition reactions during decocting (Fig. 3) [18]. Zhang et al. investigated the chemical transformation of a famous TCM preparation, Danggui Buxue Tang, an herbal decoction containing Astragali Radix and Angelicae Sinensis Radix. They found that two flavonoid glycosides, calycosin-7-*O*-*β*-d-glucoside (**32**, Fig. 4) and ononin (**33**), in Astragali Radix, could be hydrolyzed to form calycosin (**34**) and formononetin (**35**), respectively, during decocting. The hydrolysis efficiency was strongly affected by pH, temperature, and the amount of herbs [19]. Two compounds of TCM, Sanhuang Xiexin Tang (SXT) and Fuzi Xiexin Tang (FXT), have been used in China for the treatment of diseases such as diabetes for thousands of years. SXT is composed of Rhei Radix et Rhizoma, Scutellariae Radix and Coptidis Rhizoma while FXT is produced by adding another TCM, Aconiti Lateralis Radix Preparata, in SXT. Zhang et al. applied an UPLC-ESI/MS method to monitor 17 active constituents in SXT and FXT decoctions and macerations. The decoction process might enhance the dissolution of some bioactive compounds compared with the maceration process. The contents of 11 constituents [benzoylaconine (**36**), benzoylhypaconine (**37**), benzoylmesaconine (**38**), berberine (**39**), coptisine (**40**), palmatine (**41**), jatrorrhizine (**42**), aloe-emodin (**43**) and emodin (**44**), baicalin (**45**), wogonoside (**46**)] in decoctions of SXT and FXT were significantly higher than those in macerations of SXT and FXT. The β-glucuronidase in herbs could catalyze the hydrolysis of the glucuronic acid group from glycosides (baicalin and wogonoside) to transfer into aglycones [baicalein (**47**) and wogonin (**48**)]. The high temperature in the decoction process deactivated the activity of the *β*-glucuronidase and prevented the transformation of glycosides to their aglycones, which led to the discovery of the higher contents of baicalin and wogonoside in decoctions as well as the higher contents of baicalein and wogonin in macerations. The interaction between chemicals from different herbs was also observed. The diester-diterpenoid alkaloids were not detected in the decoction and maceration of FXT, but diester-diterpenoid alkaloid hypaconitine (**49**) was found in the decoction of the single herb Aconiti Lateralis Radix Preparata. The constituents of the other three herbs in FXT might promote the transformation from diester-diterpenoid alkaloids in Aconiti Lateralis Radix Preparata to other less toxic monoester-diterpenoid alkaloids, which might explain the mechanism of toxicity reduction and efficacy enhancement of TCM by formulation [20].

### Reflux extraction

Reflux extraction is more efficient than percolation or maceration and requires less extraction time and solvent. It cannot be used for the extraction of thermolabile natural products.

**Fig. 5:**
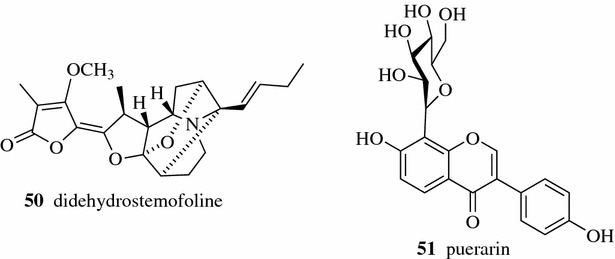
Structures of compounds **50**–**51**

Refluxing with 70% ethanol provided the highest yield of the natural bio-insecticidal, didehydrostemofoline (**50**, Fig. 5) (0.515% w/w of the extract), from *Stemona collinsiae* root among the extracts prepared by different extraction methods (sonication, reflux, Soxhlet, maceration and percolation) [21]. Zhang compared the extraction efficiency of active ingredients (baicalin (**45**, Fig. 4) and puerarin (**51**) from a TCM compound composing seven herbs with two different methods, decoction and reflux. The reflux method was found to be better than the decoction method and the highest yields of baicalin and puerarin were obtained from the reflux method with 60% ethanol as the extraction solvent [22].

### Soxhlet extraction

The Soxhlet extraction method integrates the advantages of the reflux extraction and percolation, which utilizes the principle of reflux and siphoning to continuously extract the herb with fresh solvent. The Soxhlet extraction is an automatic continuous extraction method with high extraction efficiency that requires less time and solvent consumption than maceration or percolation. The high temperature and long extraction time in the Soxhlet extraction will increase the possibilities of thermal degradation.

**Fig. 6:**
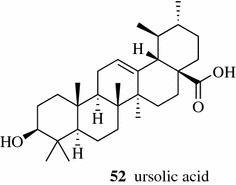
Structure of compounds **52**

Wei et al. obtained ursolic acid (**52**, Fig. 6) from the TCM Cynomorium (Cynomorii Herba) with a yield of 38.21 mg/g by Soxhlet extraction [23]. The degradation of catechins in tea was also observed in Soxhlet extraction due to the high extraction temperature applied. The concentrations of both total polyphenols and total alkaloids from the Soxhlet extraction method at 70 °C decreased compared to those from the maceration method applied under 40 °C [24, 27].

### Pressurized liquid extraction (PLE)

Pressurized liquid extraction (PLE) has also been described as accelerated solvent extraction, enhanced solvent extraction, pressurized fluid extraction, accelerated fluid extraction, and high pressure solvent extraction by different research groups. PLE applies high pressure in extraction. High pressure keeps solvents in a liquid state above their boiling point resulting in a high solubility and high diffusion rate of lipid solutes in the solvent, and a high penetration of the solvent in the matrix. PLE dramatically decreased the consumption of extraction time and solvent and had better repeatability compared to other methods.

Pressurized liquid extraction has been successfully applied by the researchers at the University of Macau and other institutes in extracting many types of natural products including saponins, flavonoids and essential oil from TCM [8, 25–27]. Some researchers believed PLE could not be used to extract thermolabile compounds due to the high extraction temperature, while others believed it could be used for the extraction of thermolabile compounds because of the shorter extraction time used in PLE. Maillard reactions occurred when PLE was used at 200 °C to extract antioxidants from grape pomace [28]. Anthocyanins are thermolabile. Gizir et al. successfully applied PLE to obtain an anthocyanin-rich extract from black carrots because the degradation rate of anthocyanins is time-dependent, and the high-temperature-short-duration PLE extraction conditions could overcome the disadvantage of high temperature employed in the extraction [29].

### Supercritical fluid extraction (SFE)

Supercritical fluid extraction (SFE) uses supercritical fluid (SF) as the extraction solvent. SF has similar solubility to liquid and similar diffusivity to gas, and can dissolve a wide variety of natural products. Their solvating properties dramatically changed near their critical points due to small pressure and temperature changes. Supercritical carbon dioxide (S-CO_2_) was widely used in SFE because of its attractive merits such as low critical temperature (31 °C), selectivity, inertness, low cost, non-toxicity, and capability to extract thermally labile compounds. The low polarity of S-CO_2_ makes it ideal for the extraction of non-polar natural products such as lipid and volatile oil. A modifier may be added to S-CO_2_ to enhance its solvating properties significantly.

**Fig. 7:**
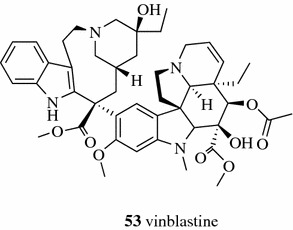
Structure of compounds **53**

Conde-Hernández extracted the essential oil of rosemary (*Rosmarinus officinalis*) by S-CO_2_ extraction, hydro distillation and steam distillation. He found that both yields of essential oil and antioxidant activity of SFC extract were higher than those from other two methods [30]. S-CO_2_ modified with 2% ethanol at 300 bar and 40 °C gave higher extracting selectivity of vinblastine (**53**, Fig. 7) (an antineoplastic drug) from *Catharanthus roseus*, which is 92% more efficient for vinblastine extraction compared to traditional extraction methods [31].

### Ultrasound assisted extraction (UAE)

Ultrasonic-assisted extraction (UAE), also called ultrasonic extraction or sonication, uses ultrasonic wave energy in the extraction. Ultrasound in the solvent producing cavitation accelerates the dissolution and diffusion of the solute as well as the heat transfer, which improves the extraction efficiency. The other advantage of UAE includes low solvent and energy consumption, and the reduction of extraction temperature and time. UAE is applicable for the extraction of thermolabile and unstable compounds. UAE is commonly employed in the extraction of many types of natural products [32, 33].

**Fig. 8:**
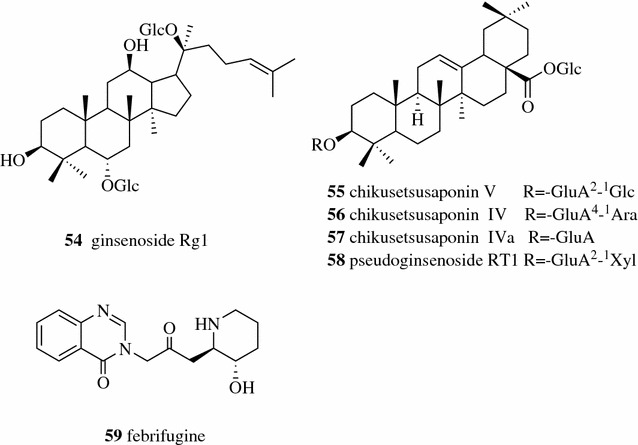
Structures of compounds **54**–**59**

Jovanović et al. achieved a higher yield of polyphenols from *Thymus serpyllum* L. by UAE at an optimized condition (50% ethanol as solvent; 1:30 solid-to-solventratio; 0.3 mm particle size and 15 min time) than maceration and heat-assisted extraction methods [13]. Wu et al. found that there was no statistically significant difference for extracting ginsenosides, including ginsenosides Rg1 (**54**, Fig. 8) and Rb1 (**7**, Fig. 3), chikusetsusaponins V (**55**), IV (**56**) and IVa **(57**), and pseudoginsenoside RT1 (**58**), from the TCM Panacis Japonici Rhizoma between UAE and reflux using 70% aqueous methanol to extract for 30 min [34]. Guo et al. found both the reflux method and UAE had the advantages of time-saving, convenient operation and high extract yield and that UAE is relatively better than reflux methods for TCM Dichroae Radix using the extract yield and content of febrifugine (**59**) as the indexes [35].

### Microwave assisted extraction (MAE)

Microwaves generate heat by interacting with polar compounds such as water and some organic components in the plant matrix following the ionic conduction and dipole rotation mechanisms. The transfers of heat and mass are in the same direction in MAE, which generates a synergistic effect to accelerate extraction and improve extraction yield. The application of MAE provides many advantages, such as increasing the extract yield, decreasing the thermal degradation and selective heating of vegetal material. MAE is also regraded as a green technology because it reduces the usage of organic solvent. There are two types of MAE methods: solvent-free extraction (usually for volatile compounds) and solvent extraction (usually for non-volatile compounds) [36, 37].

**Fig. 9:**
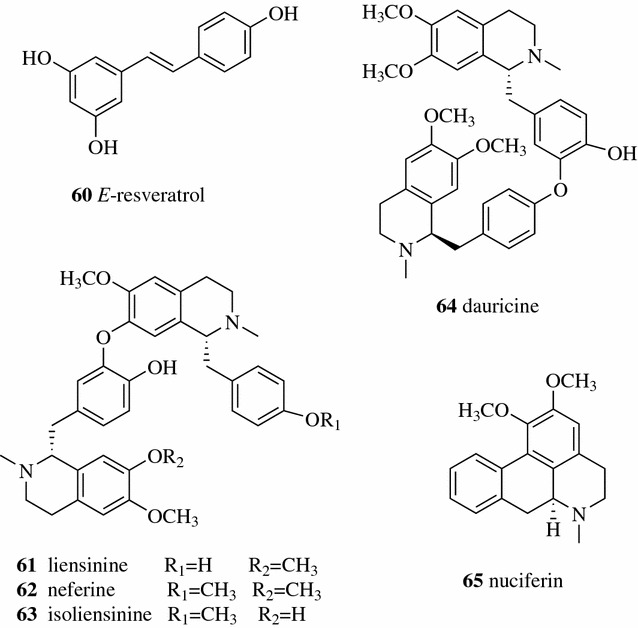
Structures of compounds **60**–**65**

Chen optimized the conditions for MAE to extract resveratrol (**60**, Fig. 9) from the TCM Polygoni Cuspidati Rhizoma et Radix (the rhizome and radix of *Polygonum cuspidatum*) by orthogonal experiment. An extraction yield of 1.76% of resveratrol was obtained from the optimized conditions as follows: extraction time 7 min, 80% ethanol, ratio of liquid to solid 25:1 (ml:g), microwave power 1.5 kw [38]. Benmoussa et al. employed the enhanced solvent-free MAE method for the extraction of essential oils from *Foeniculum vulgare* Mill. seeds at atmospheric pressure without any addition of solvent or water. The yield and aromatic profile in the enhanced solvent-free MAE extract was similar to those extracted by hydro distillation and cost only one-sixth of the time of hydro distillation [39]. Xiong et al. developed an MAE to extract five main bioactive alkaloids, liensinine (**61**), neferine (**62**), isoliensinine (**63**), dauricine (**64**), and nuciferin (**65**), from the TCM Nelumbinis Plumula (lotus plumule, the green embryo of *Nelumbo nucifera* seeds) using univariate approach experiments and central composite design. The MAE conditions was optimized as follows: 65% methanol as the extraction solvent, microwave power of 200 W and extraction time of 260 s [40, 44].

### Pulsed electric field (PEF) extraction

Pulsed electric field extraction significantly increases the extraction yield and decreased the extraction time because it can increase mass transfer during extraction by destroying membrane structures. The effectiveness of PEF treatment depends on several parameters including field strength, specific energy input, pulse number and treatment temperature. PEF extraction is a non-thermal method and minimizes the degradation of the thermolabile compounds.

Hou et al. obtained the highest yield of the ginsenosides (12.69 mg/g) by PEF using the conditions of 20 kV/cm electric field intensity, 6000 Hz frequency, 70% ethanol–water solution, and 150 l/h velocity. The yield of the ginsenosides of the PEF extraction method is higher than those of MAE, heat reflux extraction, UAE and PLE. The entire PEF extraction process took less than 1 s and much less than the other tested methods [41]. In a study of antioxidants extracted from Norway spruce bark, Bouras found that much higher phenolic content (eight times) and antioxidant activity (30 times) were achieved after the PEF treatment compared to untreated samples [42].

### Enzyme assisted extraction (EAE)

The structure of the cell membrane and cell wall, micelles formed by macromolecules such polysaccharides and protein, and the coagulation and denaturation of proteins at high temperatures during extraction are the main barriers to the extraction of natural products. The extraction efficiency will be enhanced by EAE due to the hydrolytic action of the enzymes on the components of the cell wall and membrane and the macromolecules inside the cell which facilitate the release of the natural product. Cellulose, α-amylase and pectinase are generally employed in EAE.

**Fig. 10:**
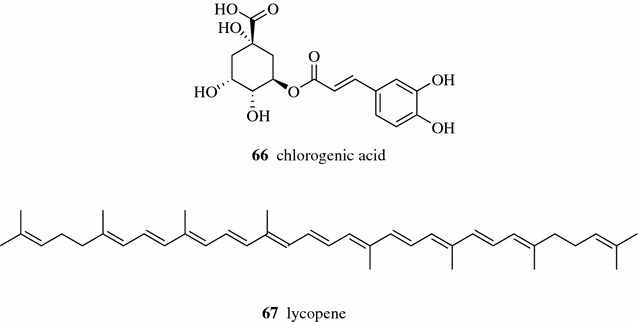
Structures of compounds **66**–**67**

Polysaccharide is one of the bioactive ingredients in the TCM Astragali Radix. Chen et al. studied the EAE of polysaccharide from the radix of *Astragalus membranaceus* using various enzymes and found that glucose oxidase offered better performance in extracting polysaccharide than the other seven enzymes tested (amyloglucosidase, hemicellulase, bacterial amylase, fungal amylase, pectinase, cellulose and vinozyme). The polysaccharide yield under the optimized EAE condition using glucose oxidase increased more than 250% compared with that from non-enzyme treated method [43]. The extraction yield of chlorogenic acid (**66**, Fig. 10) from *Eucommia ulmoides* leaves was greatly improved when using cellulase and ionic liquids [44]. Strati el al. found that carotenoid and lycopene (**67**) extraction yields from tomato waste were increased by the use of pectinase and cellulase enzymes. Compared to the non-enzyme treated solvent extraction method, sixfold and tenfold higher yields of the two target compounds were obtained in samples treated with cellulase and pectinase, respectively [45].

### Hydro distillation and steam distillation

Hydro distillation (HD) and steam distillation (SD) are commonly used methods for the extraction of volatile oil. Some natural compounds encounter decomposition in HD and SD.

The chemical composition and antibacterial activity of the primary essential oil and secondary essential oil from *Mentha citrata* were significantly affected by distillation methods. Both primary essential oil and secondary essential oil yields by HD were higher than those by SD [46, 50]. Yahya and Yunus found that the extraction time did affect the quality of the essential patchouli oil extracted. When the extraction time increased, the contents of some components decreased or increased [47].

## Separation methods

The components in the extract from above methods are complex and contain a variety of natural products that require further separation and purification to obtain the active fraction or pure natural products. The separation depends on the physical or chemical difference of the individual natural product. Chromatography, especially column chromatography, is the main method used to obtain pure natural products from a complex mixture.

### Separation based on adsorption properties

Adsorption column chromatography is widely used for the separation of natural products, especially in the initial separation stage, due to its simplicity, high capacity and low cost of adsorbents such as silica gel and macroporous resins. The separation is based on the differences between the adsorption affinities of the natural products for the surface of the adsorbents. The selection of adsorbents (stationary phase) as well as the mobile phase is crucial to achieve good separation of natural products, maximize the recovery of target compounds and avoid the irreversible adsorption of target compounds onto the adsorbents.

**Fig. 11:**
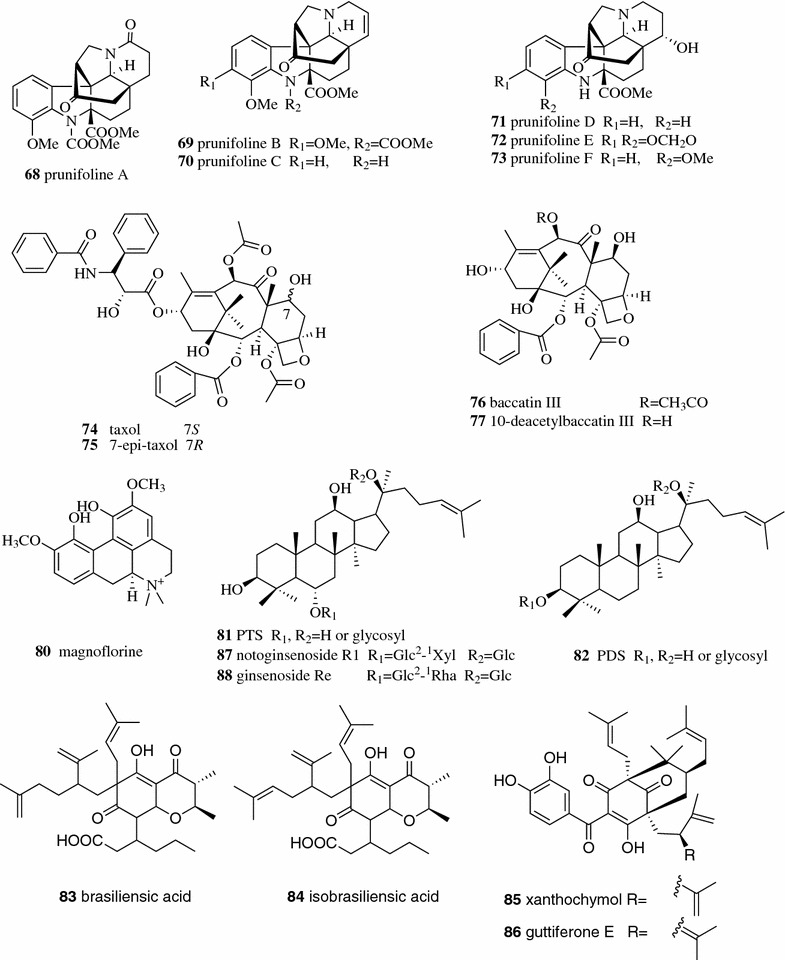
Structures of compounds **68**–**88**

Silica gel is the most widely used adsorbent in phytochemical investigation. It was estimated that nearly 90% of phytochemical separation (preparative scale) was based on silica gel. Silica gel is a polar absorbent with silanol groups. Molecules are retained by the silica gel through hydrogen bonds and dipole–dipole interactions. Thus, polar natural products are retained longer in silica gel columns than nonpolar ones. Sometimes, certain polar natural products might undergo irreversible chemisorption. The deactivation of silica gel by adding water before use or using a water-containing mobile phase will weaken the adsorption. Severe tailing may occur when separating alkaloids on silica gel, and the addition of a small amount of ammonia or organic amines such as triethylamine may reduce the tailing. Twelve alkaloids belonging to the methyl chanofruticosinate group including six new alkaloids, prunifolines A–F (**68**–**73**, Fig. 11), were obtained from the leaf of *Kopsia arborea* by initial silica gel column chromatography using gradient MeOH–CHCl_3_ as the mobile phase followed by centrifugal TLC using ammonia saturated Et_2_O–hexane or EtOAc/hexane systems as the eluent [48].

Alumina (aluminum oxide) is a strong polar adsorbent used in the separation of natural products especially in the separation of alkaloids. The strong positive field of Al^3+^ and the basic sites in alumina affecting easily polarized compounds lead to the adsorption on alumina that is different from that on silica gel. The application of alumina in the separation of natural products has decreased significantly in recent years because it can catalyze dehydration, decomposition or isomerization during separation. Zhang and Su reported a chromatographic protocol using basic alumina to separate taxol (**74**, Fig. 11) from the extract of *Taxus cuspidate* callus cultures and found the recovery of taxol was more than 160%. They found that the increase of taxol came from the isomerization of 7-*epi*-taxol (**75**) catalyzed by alumina. It was also found that a small amount of taxol could be decomposed to baccatin III (**76**) and 10-deacetylbaccatin III (**77**) in the alumina column [49]. Further investigation into the separation of taxol on acidic, neutral and basic alumina indicated that the Lewis souci and the basic activity cores on the surface of alumina induced the isomerization of 7-*epi*-taxol to taxol [50].

The structures of polyamides used in chromatography contain both acryl and amide groups. Hydrophobic and/or hydrogen bond interaction will occur in polyamide column chromatography depending on the composition of the mobile phase. When polar solvents such as aqueous solvents are used as the mobile phase, the polyamides act as the non-polar stationary phase and the chromatography behavior is similar to reversed-phase chromatography. In the contrast, the polyamides act as the polar stationary phase and the chromatography behavior is similar to normal phase chromatography. Polyamide column chromatography is a conventional tool for the separation of natural polyphenols including anthraquinones, phenolic acids and flavonoids, whose mechanisms are ascribed to hydrogen bond formation between polyamide absorbents, mobile phase and target compounds. Gao et al. studied the chromatography behavior of polyphenols including phenolic acids and flavonoids on polyamide column. It was found that the polyamide functioned as a hydrogen bond acceptor, and the numbers of phenolic hydroxyls and their positions in the molecule affected the strength of adsorption [51]. In addition to polyphenols, the separation of other types of natural products by polyamide column chromatography were also reported. The total saponins of Kuqingcha can be enriched by polyamide column chromatography, which significantly reduced the systolic pressure of SHR rat [52]. Using a mixture of dichloromethane and methanol in a gradient as the eluent, the seven major isoquinoline alkaloids in Coptidis Rhizoma including berberine (**39**), coptisine (**40**), palmatine (**41**), jatrorrhizine (**42**), columbamine (**78**), groenlandicine (**79**) (Fig. 4), and magnoflorine (**80**, Fig. 11) were separated in one-step polyamide column chromatography [53].

Adsorptive macroporous resins are polymer adsorbents with macroporous structures but without ion exchange groups that can selectively adsorb almost any type of natural products. They have been widely used either as a standalone system, or as part of a pretreatment process for removing impurities or enriching target compounds due to their advantages, which include high adsorptive capacity, relatively low cost, easy regeneration and easy scale-up. The adsorptive mechanisms of adsorptive macroporous resins include electrostatic forces, hydrogen bonding, complex formation and size-sieving actions between the resins and the natural products in solution. Surface area, pore diameter and polarity are the key factors affecting the capacity of the resins [54]. 20(*S*)-protopanaxatriol saponins (PTS) (**81**) and 20(*S*)-protopanaxadiol saponins (PDS) (**82**, Fig. 11) are known as two major bioactive components in the root of *Panax notoginseng*. PTS and PDS were successfully separated with 30 and 80% (v/v) aqueous ethanol solutions from the D101 macroporous resin column, respectively. The chromatography behaviors of PDS and PTS were close to reversed-phase chromatography when comparing the chromatographic profiles of macroporous resin column chromatography to the HPLC chromatogram on a Zorbax SB-C_18_ column [55]. Recently, Meng et al. obtained the total saponins of Panacis Japonici Rhizoma (PJRS) using D101 macroporous resin. The contents of the four major saponins, chikusetsusaponins V (**55**), IV (**56**) and IVa **(57**), and pseudoginsenoside RT1 (**58**) (Fig. 8), in the obtained PJRS was more than 73%. The PJRS served as the standard reference for quality control of Panacis Japonici Rhizoma [56]. Some researchers assumed that the principal adsorptive mechanism between macroporous resins and polyphenols was associated with the hydrogen bonding formation between the oxygen atom of the ether bond of the resin and the hydrogen atom of phenolic hydroxyl group of the phenol. The hydrogen bonding interaction force was significantly affected by the pH value of the solution [57, 58].

Silver nitrate is another useful solid support in the separation of natural products. Those natural products containing the π electrons reversibly interact with silver ions to form polar complexes. The greater the number of double bonds or aromaticity of the natural product, the stronger the complexation forms. Silver nitrate is typically impregnated on silica gel (SNIS) or alumina for separation. Several research groups reported the separation of fatty acids on SNIS [59–61]. Wang et al. reported the isolation of zingiberene from ginger oleoresin by SNIS column chromatography [62]. A pair of isomers, brasiliensic acid (**83**, Fig. 11) and isobrasiliensic acid (**84**), were separated from *Calophyllum brasiliense* by Lemos et al. on an SNIS column [63, 69]. Some research groups also applied silver nitrate in the two-phase system in high-speed counter-current chromatography (HSCCC) to improve the separation. Xanthochymol (**85**) and guttiferone E (**86**) are a pair of π bond benzophenone isomers from *Garcinia xanthochymus* by AgNO_3_-HSCCC. The elution order of the π bond isomers in this AgNO_3_-HSCCC separation is internal π bond (earlier) < terminal, which is identical to that observed from SNIS column chromatography [64].

### Separation based on partition coefficient

Partition chromatography (PC) follows the liquid–liquid extraction principle based on the relative solubility in two different immiscible liquids. In the early stage, one liquid phase was coated to a solid matrix (silica gel, carbon, cellulose, etc.) as the stationary phase and another liquid phase was employed as the mobile phase. The disadvantage of an easily removed stationary phase and unrepeatable results has led to this kind of PC being rarely used today. The bonded-phase, in which the liquid stationary phase is chemically bound to the inert support, which is used as the stationary phase overcomes those drawbacks. Commercially available alkyl such as C8 and C18, aryl, cyano and amino substituted silanes are often used as bonded phases, which are widely used to separate a variety of natural products, especially in the final purification step.

Three PTS (notoginsenoside R1 (**87**) (Fig. 11), ginsenosides Rg1 (**55**) (Fig. 8) and Re (**88**) (Fig. 11)) and two PDS [ginsenosides Rb1 (**7**) and Rd (**9**)] (Fig. 3) were well separated in a C18 column using the EtOH–H_2_O system as the mobile phase [65]. A novel polyacrylamide-based silica stationary phase was synthesized by Cai et al. and was successfully applied in the separation of galactooligosaccharides and saponins of *Paris polyphylla* with EtOH–H_2_O as the mobile phase [66].

**Fig. 12:**
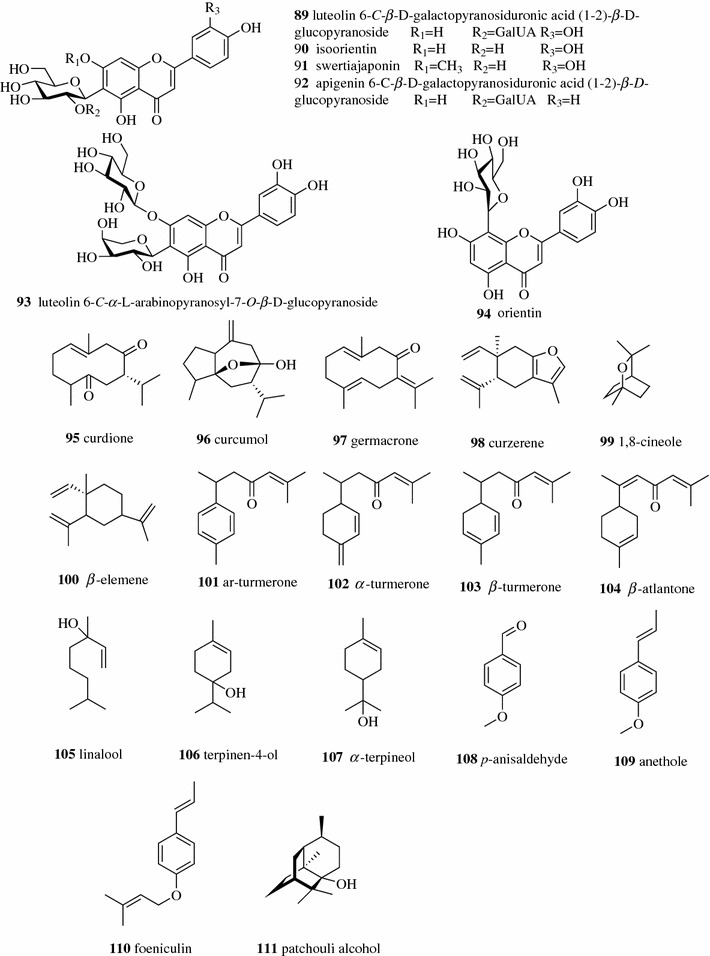
Structures of compounds **89**–**111**

Counter-current chromatography (CCC) is kind of PC that holds the liquid stationary phase by gravity or centrifugal force. CCC has rarely been used in early stages due to its poor stationary retention, long separation time and labor intensive process. CCC was significantly improved in the 1980s, however, when modern CCC, including HSCCC and centrifugal partition chromatography (CPC), were developed. The hydrodynamic CCC systems such as HSCCC have a planetary rotation movement around two rotating axes with no rotating seals, which offers a low pressure drop process. Hydrostatic CCC, e.g., centrifugal partition chromatography, uses only one rotating axis and has a series of interconnecting chambers to trap the stationary phase which offers a higher retention of the stationary phase and a higher system pressure than that of HSCCC. The high system pressure in CPC prevents the improvement of the resolution by increasing the length of the column. High performance CCC (HPCCC) represents a new generation of hydrodynamic CCC and works in the same way as HSCCC, but with a much higher g-level. The HPCCC instruments generate more than 240 g, while early HSCCC equipment gave g-levels of less than 80 g. HPCCC shortens the separation time to less than an hour compared to several hours in previous HSCCC and can achieve at least ten times the throughput of an HSCCC instrument [67]. Compared to the conventional column separation method using a solid stationary phase, both hydrostatic and hydrodynamic CCC systems offer some advantages including the elimination of irreversible adsorption and peak tailing, high loading capacity, high sample recovery, minimal risk of sample denaturation and low solvent consumption. The limitation of CCC is that it only separates the compounds in a relatively narrow polarity window. Over the past 20 years, HSCCC, HPCCC and CPC attracted great attention in separation science and have been widely used in the separation of natural products. Tang et al. developed an HSCCC method using a two-phase solvent system comprising ethyl acetate–*n*-butanol–ethanol–water (4:2:1.5:8.5, v/v/v/v) to separate six flavone *C*-glycosides (**89**–**94**, Fig. 12), including two novel compounds from *Lophatherum gracile* [68]. HSCCC, HPCCC and CPC have also been successfully applied in the separation of volatile oil, which is difficult to separate via conventional column chromatography. Six volatile compounds (curdione (**95**), curcumol (**96**), germacrone (**97**), curzerene (**98**), 1,8-cineole (**99**) and *β*-elemene (**100**)) were isolated by CPC from the essential oil of *Curcuma wenyujin* using a nonaqueous two-phase solvent system consisting of petroleum ether–acetonitrile–acetone (4:3:1 v/v/v) [69]. Four major sesquiterpenoids (ar-turmerone (**101**), *α*-turmerone (**102**), *β*-turmerone (**103**), and *E*-atlantone (**104**)) with similar structures were separated from the essential oil of *Curcuma longa* in a single HSCCC run using a two-phase solvent system composed of *n*-heptane–ethyl acetate–acetonitrile–water (9.5/0.5/9/1, v/v) and each compound achieved over 98% purity [70]. Linalool (**105**), terpinen-4-*ol* (**106**), *α*-terpineol (**107**), *p*-anisaldehyde (**108**), anethole (**109**) and foeniculin (**110**) were successfully isolated from the essential oil of *Pimpinella anisum* by HPCCC using a stepwise gradient elution [71]. Li et al. developed a CPC method for the separation of patchouli alcohol (**111**) with a nonaqueous ether–acetonitrile (1:1, v/v) solvent system. More than 2 g of patchouli alcohol with over 98% purity were isolated from 12.5 g of essential oil over a 240 ml column [72]. The large volume (several liters) column has been adopted in commercial hydrostatic CCC and hydrodynamic CCC equipment for pilot/industrial scale separation. Few reports could be obtained due to commercial confidentiality. It is difficult to judge whether hydrostatic or hydrodynamic CCC is better for industrial applications. Users might select different types of CCC instrument for different purposes. When the stationary phase is poorly retained in hydrodynamic CCC due to high viscosity and small density differences between the mobile and stationary phases, the hydrostatic CCC is more practical than hydrodynamic CCC because the retention of the stationary phase of hydrostatic CCC is less sensitive to the physical properties of liquid systems and will have a higher retention of the stationary phase. When the stationary phase is well retained in hydrodynamic CCC, higher separation efficiency will be obtained from hydrodynamic CCC than from hydrostatic CCC with the same liquid system and similar column volumes because hydrostatic CCC has relatively low partition efficiency due to a limited degree of mixing, and the hydrodynamic system provides efficient mixing to yield a high partition efficiency.

### Separation based on the molecular size

The separation of natural products by membrane filtration (MF) or gel filtration chromatography (GFC) is based on their molecular sizes.

#### Membrane filtration (MF)

In MF, the semipermeable membrane allows smaller molecules to pass through and retains the larger molecules. MF of natural products could be characterized as microfiltration, ultrafiltration, and nanofiltration based on the pore size of the membrane applied.

**Fig. 13:**
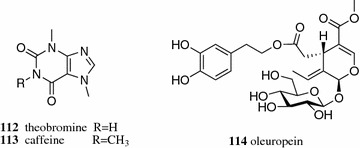
Structures of compounds **112**–**114**

Membrane filtration has been a powerful tool for the concentration, clarification and removal of impurities in the lab, as well as in the food and pharmaceutical industries. The contents of total phenols (338%), chlorogenic acid (**66**) (Fig. 10) (483%), theobromine (**112**, Fig. 13) (323%), caffeine (**113**) (251%), condensed tannins (278%) and saponins (211%) in the aqueous extract of *Ilex paraguariensis* were significantly increased by nanofiltration [73, 80]. Coupling membrane filtration is applied when a single membrane filtration step is not satisfactory. A sequence of microfiltration, ultrafiltration and nanofiltration was applied in the isolation of bioactive components from olive leaf extract. Microfiltration followed by ultrafiltration removed the impurities larger than 5 kDa. Nanofiltration recovered the antioxidative and antibacterial polyphenols and flavonoids, and the content of the major component, oleuropein (**114**), in the nanofiltration retentate was concentrated approximately ten times [74].

#### Gel filtration chromatography (GFC)

Gel filtration chromatography is also known as gel permeation chromatography or size exclusion chromatography. The small molecules have a longer retention time in GFC than large molecules.

Sephadex is formed by cross-linking dextran, and the G-types of Sephadex were used for the separation of hydrophilic compounds such as peptides [75], oligosaccharides and polysaccharides [76].

**Fig. 14:**
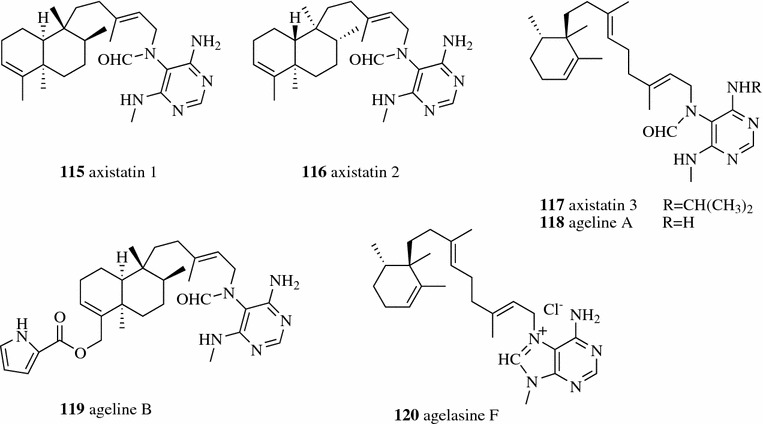
Structures of compounds **115**–**120**

Sephadex LH20, a hydroxypropylated derivative of Sephadex G25, has both hydrophobic and hydrophilic natures. An adsorption mechanism was also involved in separation using Sephadex LH-20. Sephadex LH-20 can be used for the separation of a wide variety of natural products in either an aqueous or non-aqueous solvent system. The feruloylated arabinoxylan oligosaccharides of perennial cereal grain intermediate wheat were well separated by Sephadex LH-20 using 100% water as the mobile phase [77]. Three new pyrimidine diterpenes, axistatins 1–3 (**115**–**117**, Fig. 14) along with three known formamides (**118**–**120**) were isolated from the anti-cancer active CH_2_Cl_2_ fraction of *Agelas axifera* over Sephadex LH-20 columns with a series of solvent systems [CH_3_OH, CH_3_OH–CH_2_Cl_2_ (3:2), hexane–CH_3_OH–2-propanol (8:1:1), hexane–toluene–CH_2_Cl_2_–EtOH (17:1:1:1) and exane–EtOAc–CH_3_OH (4:5:1)], followed by purification using Prep-HPLC [78, 85, 87].

Polyacrylamide (bio-gel P) [79] and cross-linked agarose [80] were also used in the separation of natural products.

### Separation based on ionic strength

Ion-exchange chromatography (IEC) separates molecules based on the differences in their net surface charge. Some natural products, such as alkaloids and organic acids possessing a functional group capable of ionization, might be separated by IEC. The charged molecules could be caught and released by ion-exchange resin by changing the ionic strength of the mobile phase (e.g., changing pH or salt concentration). Cation ion-exchange resins were used for the separation of alkaloids, while the anion ion-exchange resins were used for the separation of natural organic acids and phenols.

**Fig. 15:**
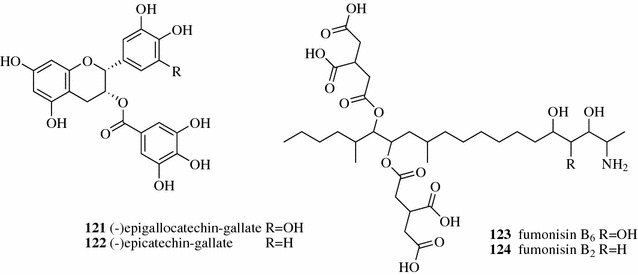
Structures of compounds **121**–1**24**

The positively charged anthocyanins were separated from the neutral polyphenolic compounds in the XAD-7 treated *Actinidia melanandra* fruit (kiwifruit) extract using Dowex 50WX8 cation ion-exchange resin [81]. Feng and Zhao used semi-preparative chromatography to separate (−)epigallocatechin-gallate [**121**, Fig. 15)] and (−)epicatechin-gallate (**122**) in tea crude extract with polysaccharide-based weakly acidic gel CM-Sephadex C-25 [82]. A new alkaloid, fumonisin B_6_ (**123**), along with a known alkaloid, fumonisin B_2_ (**124**), was isolated by IEC over Strata X-C mixed-mode RP-cation-exchange resin followed by reverse-phase chromatography from the fungus *Aspergillus niger* NRRL 326 cultures extract [83].

### Other modern separation techniques

#### Molecular distillation (MD)

**Fig. 16:**
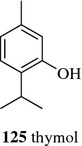
Structure of compounds **125**

Molecular distillation separates the molecular by distillation under vacuum at a temperature far below its boiling point. It is a suitable distillation method for separating thermosensitive and high-molecular-weight compounds. Borgarello et al. obtained a thymol (**125**, Fig. 16) enrichment fraction from oregano essential oil by molecular distillation modeled by artificial neural networks. The obtained fraction had antioxidant properties and could stabilize the sunflower oil [84]. Three kinds of phthalates were effectively removed from sweet orange oil by molecular distillation under the optimal conditions (evaporation temperature of 50 °C, evaporator pressure of 5 kPa and a feed flow rate of 0.75 ml/min) [85].

#### Preparative gas chromatography (Prep-GC)

Gas chromatography (GC) with high separation efficiency and fast separation and analysis makes it potentially the ideal preparative method for the separation of volatile compounds. The injection port, column, split device and trap device of GC equipment must be modified for preparative separation due to a lack of commercial Prep-GC [86].

**Fig. 17:**
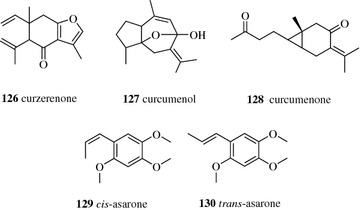
Structures of compounds **126**–**130**

Five volatile compounds, namely, curzerene (**98**) (6.6 mg), *β*-elemene (**100**, Fig. 12) (5.1 mg), curzerenone (**126**) (41.6 mg), curcumenol (**127**) (46.2 mg), and curcumenone (**128**) (21.2 mg) (Fig. 17), were separated from the methanol extract of Curcuma Rhizome by Prep-GC over a stainless steel column packed with 10% OV-101 (3 m × 6 mm, i.d.) after 83 single injections (20 μl) [87]. Prep-GC was also applied for the separation of natural isomers. A total of 178 mg of *cis*-asarone (**129**) and 82 mg of *trans*-asarone (**130**) were obtained from the essential oil of *Acorus tatarinowii* after 90 single injections (5 μl) on the same column as above [88]. Prep-GC has become an important separation method for natural volatile compounds; however, a heavier sample load and the large-diameter preparative column employed decreased the efficiency [89]. Meanwhile, the disadvantages of Prep-GC, including the lack of commercial Prep-GC equipment, consumption of a large volume of carrier gas, the decomposition of thermolabile compounds under high operation temperature, the difficulties of fraction collection, and low production, still restrict the usage of Prep-GC.

#### Supercritical fluid chromatography (SFC)

SFC uses supercritical fluid as the mobile phase. SFC integrates the advantages of both GC and liquid chromatography (LC) as the supercritical fluids possess properties of high dissolving capability, high diffusivity and low viscosity, which allows rapid and efficient separation. Thus, SFC can use a longer column and smaller particles of the stationary phase than HPLC, which provides greater numbers of theoretical plates and better separation. SFC can be used for the separation of non-volatile or thermally labile compounds to which GC is not applicable. SFC systems are compatible with a wide range of different detectors including those used in LC and GC systems. The polarity of the widely used mobile phase, S-CO_2_, in SFC is close to the polarity of hexane, with the result that SFC was used for the separation of non-polar natural products such as fatty acids, terpenes and essential oils for many years. Eluent modifiers such methanol and acetonitrile enhance the elution strength, which is increasing the interest in separating polar natural products by SFC [90–92].

**Fig. 18:**
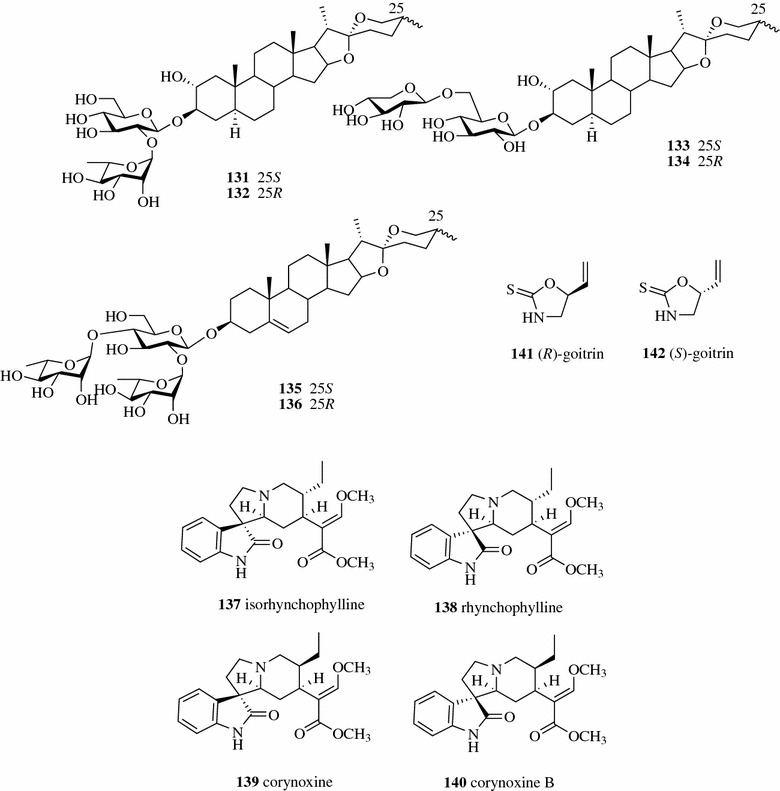
Structures of compounds **131**–**142**

Zhao et al. successfully separated three pairs of 25 *R*/*S* diastereomeric spirostanol saponins (**131**–**136**, Fig. 18) from the TCM Trigonellae Semen (the seed of *Trigonella foenum*-*graecum*) on two CHIRALPAK IC columns coupled in tandem [93]. Yang et al. applied SFC for the preparative separation of two pairs of 7-epimeric spiro oxindole alkaloids (**137**–**140**) from stems with hooks of *Uncaria macrophylla* (a herbal source for TCM Uncariae Ramulus Cum Uncis) on a Viridis Prep Silica 2-EP OBD column using acetonitrile containing 0.2% DEA modified S-CO_2_. The non-aqueous mobile phase used in SFC prevented the tautomerization of the separated spiro oxindole alkaloids [94]. SFC is also applied in the separation of natural enantiomers. (*R*,*S*)-goitrin (**141**–**142**) is the active ingredient of TCM Isatidis Radix. The chiral separation of (*R*) and (*S*) goitrins was successfully achieved by prep-SFC on a Chiralpak IC column using acetonitrile as the organic modifier [95].

#### Molecular imprinted technology

Molecular imprinted technology has been an attractive separation method in the last decade due to its unique features, which include high selectivity, low cost and easy preparation. Many complementary cavities with the memory of size, shape, and functional groups of the template molecules are generated when the template molecules are removed from the molecular imprinted polymer (MIP). Thus, the template molecule and its analogs will have the specific recognition and selective adsorption for the MIP. MIPs have been widely used in the separation of natural products or as solid-phase extraction sorbents for sample preparation of herbal materials to enrich the minor compounds.

**Fig. 19:**
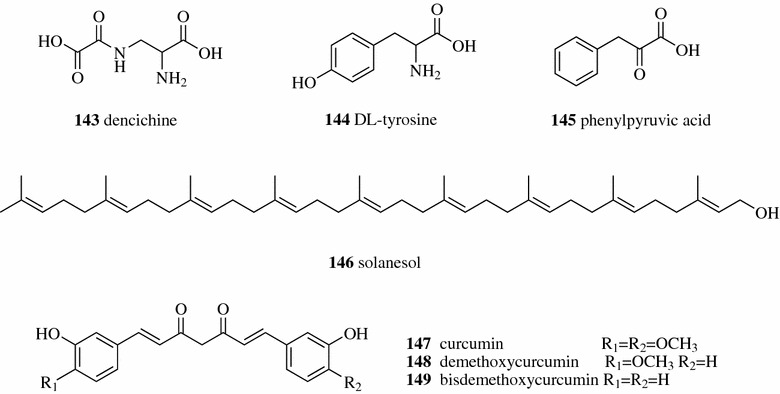
Structures of compounds **143**–**149**

Ji et al. developed multi-template molecularly imprinted polymers using DL-tyrosine and phenylpyruvic acid as the template molecules to separate dencichine (**143**, Fig. 19) from the water extract of *Panax notoginseng*. Both dencichine and the template molecule of DL-tyrosine (**144**) contain an amino (NH_2_) group and a carboxylic acid (COOH) group, and the other template molecule, phenylpyruvic acid (**145**), has an α-keto acid (COCOOH) group that can also be found in the structure of dencichine [96]. Ma et al. developed a preparative separation method to separate solanesol (**146**) from tobacco leaves by flash chromatography based on MIP. The MIP was prepared with methyl methacrylate as the monomer, solanesol as the template molecule and ethylene glycol dimethacrylate as the crosslinker by a suspension polymerization method. A total of 370.8 mg of solanesol with 98.4% purity was separated from the extract of tobacco leaves with a yield of 2.5% of the dry weight of tobacco leaves [97]. You et al. used the thermo-responsive magnetic MIP to separate the three major curcuminoids, curcumin (**147**), demethoxycurcumin (**148**), and bisdemethoxycurcumin (**149**), from the TCM Curcumae Longae Rhizoma (the rhizome of *Curcuma longa*). The designed thermo-responsive magnetic MIP showed good imprinting factor for curcuminoids in a range between 2.4 and 3.1, thermo-responsiveness [lower critical solution temperature at 33.71 °C] and rapid magnetic separation (5 s) [98].

#### Simulated moving bed chromatography

Simulated moving bed (SMB) chromatography uses multiple columns with stationary phases (bed). The countercurrent movement of the bed is simulated through rotary valves, which periodically switch the inlet (feed and eluent) and outlet (extract and raffinate). The SMB process is a continuous separation method and a powerful tool for the large-scale separation of natural products with the advantage of lower solvent consumption over a shorter period of time.

**Fig. 20:**
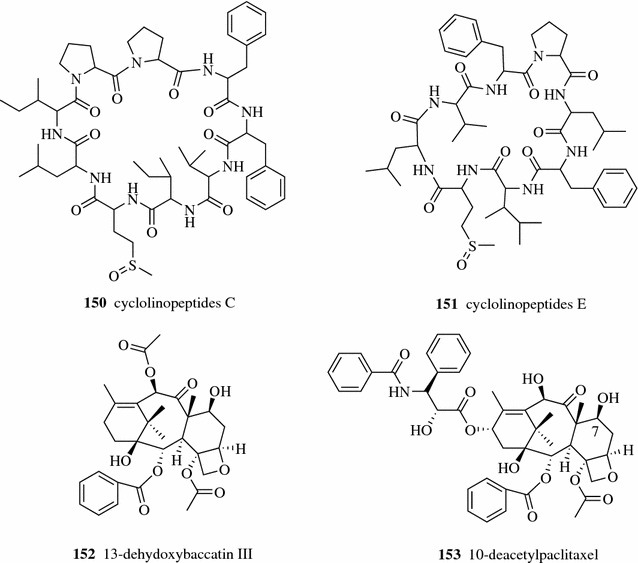
Structures of compounds **150**–**153**

Two cyclopeptides, cyclolinopeptides C and E (**150–151**, Fig. 20), were obtained from flaxseed oil using a three zone SMBC with eight preparative HPLC normal phase spherical silica gel columns and using absolute ethanol as the desorbent [99]. Kang et al. developed a tandem SMB process consisting of two four-zone SMB units in a series with the same adsorbent particle sizes in Ring I and Ring II to separate paclitaxel (taxol, **74**) (Fig. 11), 13-dehydroxybaccatin III (**152**), and 10-deacetylpaclitaxel (**153**). Paclitaxel was recovered in the first SMB unit while 13-dehydroxybaccatin III and 10-deacetylpaclitaxel were separated in the second SMB unit [100]. Mun enhanced this SMB chromatography method by using different particle sizes adsorbent in Ring I and Ring II [101]. Supercritical fluids can also be used as the desorbent in SMB chromatography. Liang et al. successfully applied supercritical carbon dioxide with ethanol as the desorbent for a three-zone SMB to separate resveratrol (**60**) (Fig. 9) and emodin (**44**) (Fig. 4) from a crude extract of the TCM Polygoni Cuspidati Rhizoma et Radix [102].

#### Multi-dimensional chromatographic separation

The components in the extract subjected to separation were complex, and generally, no pure compound will be separated in one column chromatography. Multi-dimensional separation based on the solid phase extraction and coupling of multiple columns with different stationary phases greatly improves the separation efficiency. With more commercial multiple dimensional separation equipment entering the market, the separation of natural products is becoming more rapid, efficient and automated.

**Fig. 21:**
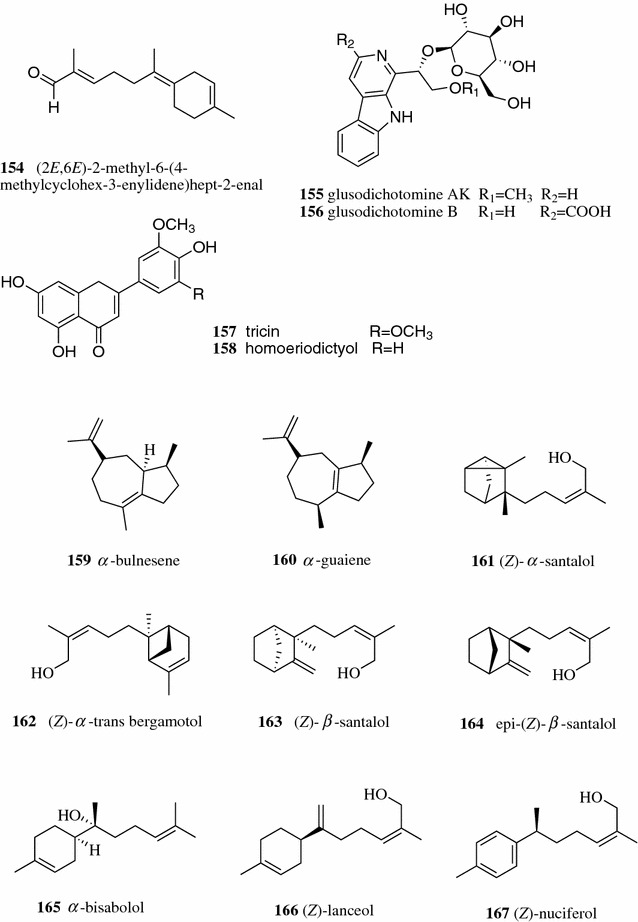
Structures of compounds **154**–**167**

Usually, the target compound was enriched by first dimensional separation and purified by last dimensional separation. Multi-dimensional separation can be achieved using the same type separation equipment (LC or GC) or different types of equipment (GC and LC). A novel volatile compound, (2*E*,6*E*)-2-methyl-6-(4-methylcyclohex-3-enylidene)hept-2-enal (**154**), was purified by a three-dimensional prep-GC from wampee essential oil [103]. Five antioxidant compounds, including two alkaloids [glusodichotomine AK (**155**) and glusodichotomine B (**156**)] and three flavonoids [tricin (**157**), homoeriodictyol (**158**) (Fig. 21), and luteolin (**3**) (Fig. 1)], were separated using a two-dimensional HPLC (RP/HILIC) method from *Arenaria kansuensis* on a RP-C18HCE and a NP-XAmide preparative columns [104]. Sciarrone et al. exploited the separation of sesquiterpenes in patchouli essential oil by three dimensional Prep-GC. Patchouli alcohol (**111**, Fig. 12) (496 μg) was separated in the first dimension on a poly(5% diphenyl/95% dimethylsiloxane) column, and 295 μg of *α*-bulnesene (**159**) was from a second column coated with high molecular weight polyethylene glycol as well as 160 μg *α*-guaiene (**160**) from the third dimension on an ionic-liquid based column (SLB-IL60) [105]. Pantò et al. applied two three-dimensional approaches (GC–GC–GC and LC–GC–GC) to separate the sesquiterpene alcohols [(*Z*)-*α*-santalol (**161**), (*Z*)-*α*-*trans* bergamotol (**162**), (*Z*)-*β*-santalol (**163**), *epi*-(*Z*)-*β*-santalol (**164**), *α*-bisabolol (**165**), (*Z*)-lanceol (**166**), and (*Z*)-nuciferol (**167**)] from the sandalwood essential oil. They found that the first dimensional separation using LC reduced the sample complexity and increased the productivity of low-concentration components [106].

## Summary

Natural products have contributed to drug development over the past few decades and continue to do so. The lab-intensive and time-consuming of extraction and isolation processes, however, have hindered the application of natural products in drug development. As technology continues to develop, more and more new automatic and rapid techniques have been created to extract and separate natural products, which might reach the requirement of high-throughput screening.

Regarding extraction, reflux extraction is the most commonly employed technique for preparative separation. The modern extraction methods, also regarded as green extraction methods, including UAE, MAE, SFE and PLE, have also been the subject of increased attention in recent years due to their high extraction yields, selectivity, stability of the target extracts and process safety merits. Some of those green methods have become routine sample preparation methods for analytical purposes.

Regarding isolation, the development of novel packing material could enhance the efficiency of isolation, which should be researched further. The hyphenation of chromatographic and spectroscopic or spectrometric techniques with the aim of elucidating structures without the need for isolation, such as LC-NMR and LC–MS, is a useful dereplication tool for searching for novel natural products. Although the isolation of pure natural products from complex mixtures remains challenging and we are far from one-step isolation procedures, the application of more selective methods from extraction to fractionation and purification will speed up the time from collecting biological material to isolating the final purified compound.

In conclusion, there is a clear and increasing interest in the extraction and isolation of natural products and their advantageous applications. These specific applications are also conditioning the employed extraction methods and novel stationary phases and mobile phases to be used by these techniques. It is thus expected that these trends will be maintained in the near future as they are mostly motivated by emerging consumer demands and by safety, environmental and regulatory issues.

## Abbreviations

CCC: counter-current chromatography
CPC: centrifugal partition chromatography
FXT: Fuzi Xiexin Tang
GC: gas chromatography
GFC: gel filtration chromatography
HD: hydro distillation
HPCCC: high performance counter-current chromatography
HPLC: high-performance liquid chromatography
HSCCC: high-speed counter-current chromatography
IEC: ion-exchange chromatography
LC: liquid chromatography
MAE: microwave assisted extraction
MD: molecular distillation
MF: membrane filtration
MIP: molecular imprinted polymer
PC: partition chromatography
PDS: 20(S)-protopanaxadiol saponins
PEF: pulsed electric field
PLE: pressurized liquid extraction
PJRS: total saponins of Panacis Japonici Rhizoma
Prep-GC: preparative gas chromatography
PTS: 20(S)-protopanaxatriol saponins
S-CO2: supercritical carbon dioxide
SD: steam distillation
SF: supercritical fluid
SFC: supercritical fluid chromatography
SFE: supercritical fluid extraction
SMB: simulated moving bed
SNIS: impregnated on silica gel
SXT: Sanhuang Xiexin Tang
TCM: traditional Chinese medicine
UAE: ultrasonic-assisted extraction
